# Anti-Inflammatory Effects of Ellagic Acid on Acute Lung Injury Induced by Acid in Mice

**DOI:** 10.1155/2013/164202

**Published:** 2013-02-27

**Authors:** Daniely Cornélio Favarin, Maxelle Martins Teixeira, Ednéia Lemos de Andrade, Claudiney de Freitas Alves, Javier Emilio Lazo Chica, Carlos Artério Sorgi, Lúcia Helena Faccioli, Alexandre Paula Rogerio

**Affiliations:** ^1^Laboratório de ImunoFarmacologia Experimental (LIFE), Universidade Federal do Triângulo Mineiro (UFTM), Departamento de Clínica Médica, Instituto de Ciências da Saúde, Uberaba MG, Brazil; ^2^Departamento de Farmacologia, Universidade Federal de Santa Catarina, Florianópolis, SC, Brazil; ^3^Departamento de Análises Clínicas, Toxicológicas e Bromatológicas, Faculdade de Ciências Farmacêuticas de Ribeirão Preto, Universidade de São Paulo (USP), Ribeirão Preto, SP, Brazil; ^4^Departamento de Clínica Médica, Universidade Federal do Triângulo Mineiro, Rua Manoel Carlos 162, 38025-380 Uberaba, MG, Brazil

## Abstract

Acute lung injury (ALI) is characterized by alveolar edema and uncontrolled neutrophil migration to the lung, and no specific therapy is still available. Ellagic acid, a compound present in several fruits and medicinal plants, has shown anti-inflammatory activity in several experimental disease models. We used the nonlethal acid aspiration model of ALI in mice to determine whether preventive or therapeutic administration of ellagic acid (10 mg/kg; oral route) could interfere with the development or establishment of ALI inflammation. Dexamethasone (1 mg/kg; subcutaneous route) was used as a positive control. In both preventive and therapeutic treatments, ellagic acid reduced the vascular permeability changes and neutrophil recruitment to the bronchoalveolar lavage fluid (BALF) and to lung compared to the vehicle. In addition, the ellagic acid accelerated the resolution for lung neutrophilia. Moreover, ellagic acid reduced the COX-2-induced exacerbation of inflammation. These results were similar to the dexamethasone. However, while the anti-inflammatory effects of dexamethasone treatment were due to the reduced activation of NF-**κ**B and AP-1, the ellagic acid treatment led to reduced BALF levels of IL-6 and increased levels of IL-10. In addition, dexamethasone treatment reduced IL-1**β**. Together, these findings identify ellagic acid as a potential therapeutic agent for ALI-associated inflammation.

## 1. Introduction

Acute lung injury (ALI) and acute respiratory distress syndrome (ARDS) are life-threatening syndromes that cause high morbidity and mortality [1]. Epidemiologic data have revealed that the incidence of ALI/ARDS varies widely by geographic location. The incidence ranges from 64.2 to 78.9 cases/100.000 person-years in the USA, and in Northern Europe, it is of 17 cases/100.000 person-years [2]. ALI is characterised by alveolar edema and uncontrolled neutrophil migration to the lung. The subsequent neutrophil activation leads to tissue damage via the release of proteases, oxidants, and cationic peptides [3]. Through the recent use of molecular and cellular assays and knockout animals, considerable progress has been made towards the understanding of the genetic, tissue-specific, and immunological factors that contribute to the development of ALI pathophysiology. However, no specific therapies are available, and the disease outcome has yet to be improved by pharmacologic treatments [4]. Ventilation with lower tidal volumes is the only method that has shown a level of benefit [5]. Thus, the identification of new molecules that can modulate ALI-associated inflammation is highly desirable and is a significant goal of pharmaceutical companies.

Recent evidence has suggested that certain compounds are effective in inhibiting neutrophil function and infiltration, and such inhibition is desirable for the treatment of patients with ALI and sepsis. Natural products and their derivatives (secondary metabolites) are the most common sources of these drugs. Many plant-derived secondary metabolites are capable of directly modulating inflammation by altering the production and activity of second messengers, the expression of transcription factors, and the expression of key proinflammatory molecules [6–8]. In addition, they can provide relief from symptoms that is comparable to that obtained from allopathic medicines. Ellagic acid, a polyphenol, is present in several fruits, such as grapes, strawberries, pomegranates, and walnuts, as well as several medicinal plants [9–11]. Ellagic acid has exhibited antioxidant [12], anticancer [13], antiallergic [14], and anti-inflammatory [11, 15] activities, among others. Interestingly, ellagic acid, as well as extracts that are rich in ellagic acid, has demonstrated significant effects on the airways. In this context, *Lafoensia pacari* Jaume St. Hilaire (Lythraceae) extracts and ellagic acid reduced most of the phenotypes of experimental ovalbumin-induced allergic airway inflammation [16], and *Punica granatum* (Lythraceae) fruit extract reduced the airway inflammation during LPS-induced ALI in mice [17].

Here, we used the nonlethal acid-initiated experimental model of ALI to determine whether preventive or therapeutic administration of ellagic acid could interfere with the development and establishment of ALI-associated inflammation. In this study, ellagic acid demonstrated potent anti-inflammatory effects, accelerated the resolution of inflammation, and decreased the exacerbation of the inflammation process caused by selective COX-2 inhibition. Taken together, these results suggest the potential of ellagic acid as a candidate for the treatment of ALI-associated inflammation. 

## 2. Methods

### 2.1. Animals

All animal care and procedures used in this study were in compliance with the guidelines on the Use of Animals of the UFTM Ethics Committee (Protocol no. 162), which follow the NIH “Principles of Laboratory Animal Care” Publication no. 85–23. The experiments were conducted using female BALB/c mice (5–7 weeks old and weighing 20–25 g) that were kept in controlled temperature (22 ± 2°C) and humidity (45%–55%) under a 12:12 h light-dark cycle (lights on 07:00 h). 

### 2.2. Acid-Initiated Acute Lung Injury

The mice were anesthetised with ketamine (50 mg/kg) and xylazine (8 mg/kg), and hydrochloric acid (0.1 N HCl, pH 1.5, 50 *µ*L) was intratracheally instilled into the left lung via a 24-gauge angiocatheter. At 12, 48, and/or 72 h following acid-initiated acute lung injury bronchoalveolar lavage fluid (BALF) and lung tissue were harvested [18]. One group of animals was instilled with saline into the left lung (the control group).

### 2.3. Treatment with Ellagic Acid

The treatment of animals with ellagic acid was carried out as described by Rogerio et al. [15]. Once ellagic acid demonstrates poor solubility in water, the treatment in each animal was carried out with a suspension of ellagic in water at dose 10 mg/kg [15]. The suspension was homogenized with the syringe used in the oral administration before each animal treatment. To study the preventive anti-inflammatory effects, ellagic acid (10 mg/kg) [15] or a vehicle control (water) was given daily by oral gavage (30 minutes prior and 24, 47, and/or 71 h following the intratracheal acid instillation). In a second cohort, the mice were therapeutically treated with ellagic acid or the vehicle by oral gavage (2, 24, 47, and/or 71 h following the acid-initiated injury). In a third cohort, the ellagic acid or the vehicle was administered during the resolution phase after the peak of inflammation (12 h) at 24, 47, and/or 71 h following acid-initiated acute lung injury. In a fourth cohort, the animals were pretreated with a selective inhibitor of cyclooxygenase-2 (COX-2) (Celebra, 10 mg/kg; intraperitoneal route) 30 min prior to the intratracheal acid instillation [18, 19] and then treated with the ellagic acid or the vehicle (2, 24, and 47 h after the acid-initiated injury). As a positive control, the mice were treated with dexamethasone as described earlier (1 mg/kg, s.c. injection) [15].

### 2.4. Evaluation of the Leukocyte Influx into the Bronchoalveolar Space

At 12, 48, and/or 72 h following the acid-initiated acute lung injury, the mice were euthanised by sodium pentobarbital overdose (70 mg/kg, intraperitoneal), and the BALF and lung were collected. A polyethylene cannula was introduced into the trachea. BALF was performed with 1 mL of phosphate-buffered saline (PBS) plus 0.6 mm ethylenediamine tetraacetic acid (EDTA) and placed on ice. The total cell and differential leukocyte counts were made according to Rogerio et al. [15]. Following centrifugation (400 ×g, 5 min, 4°C), the supernatants of the BALF were collected and stored at −80°C for subsequent cytokine determination.

### 2.5. Endothelial Permeability

Evans Blue dye was utilised as a marker for endothelial barrier function. The animals were injected with Evans Blue dye (30 mg/kg) via the left retro-orbital plexus 2 h prior to euthanasia. The BALF was collected at 12 h following the acid-initiated acute lung injury, and the extravasation of the dye was quantified by spectrophotometry (absorbance at 650 nm) [20, 21].

### 2.6. Measurement of IL-6, KC, IL-1*β*, and IL-10 Levels

IL-6, KC, IL-1*β*, and IL-10 levels were assayed by ELISA according to the manufacturer's instructions (R&D Systems, Minneapolis, MN, USA). Sensitivities were >10 pg/mL.

### 2.7. Statistical Analysis

The data were reported as the means ± SEM. The means from the different treatments in each individual experiment were compared by ANOVA. When significant differences were identified, the individual comparisons were subsequently made with Tukey's test. Values of  *P* < 0.05  were considered statistically significant.

## 3. Materials

Ellagic acid (Sigma-Aldrich, MO, USA), Dexamethasone (Decadron, Laboratório Teuto Brasileiro, GO, BRA), Celecoxib (Celebra, Pfizer Pharmaceuticals LLC, SP, BRA), Evans Blue (Vetec Química Fina Ltda, RJ, BRA), HCl (Vetec Química Fina Ltda, RJ, BRA), xylazine (Hertape Calier Saude Animal S/A, MG, BRA), and ketamine (Cristália-Produtos Químicos Farmacêuticos Ltda, SP, BRA) were purchased. 

## 4. Results

### 4.1. The Preventive Effect of Ellagic Acid on Leukocyte Recruitment to the BALF

**Figure 1:**
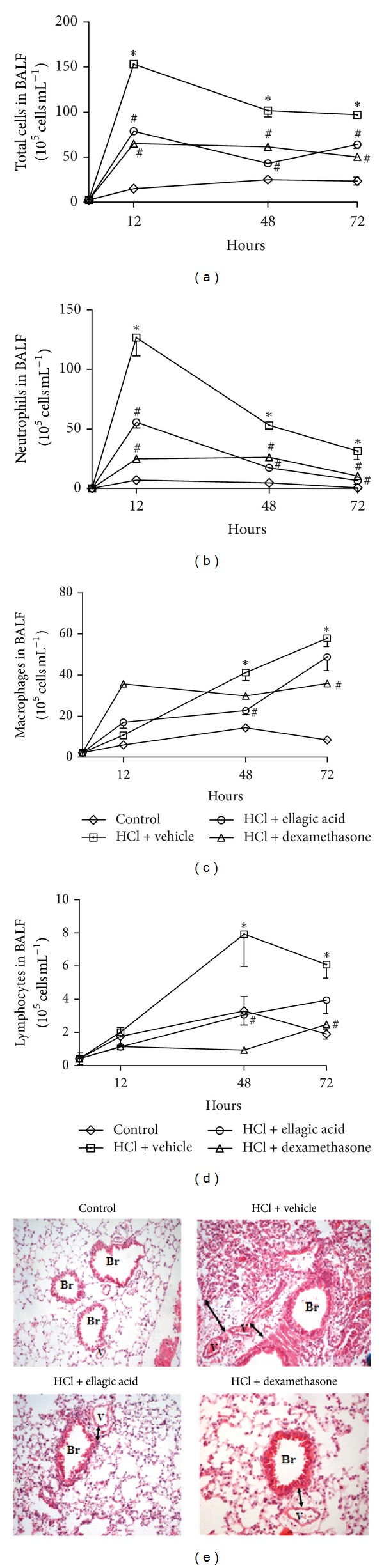
Ellagic acid prevents the development of airway inflammatory responses in the experimental model acid-initiated acute lung injury. Mice received ellagic acid (10 mg/kg, p.o.), dexamethasone (1 mg/kg, s.c.), or vehicle (water, p.o.) 30 minutes prior and/or after (24, 47, and/or 71 h) the acid intratracheal instillation into the left lung (see Methods). The kinetics of inflammation was determined at 12, 48, and 72 hours after acid-initiated acute lung injury. (a) Total cells, (b) neutrophils, (c) macrophages, and (d) lymphocytes were evaluated in the BALF. One group of animals received saline intratracheal instillation (control group). (e) Lung tissue inflammation was assessed by hematoxylin and eosin staining (H&E, original magnification, 200x). Results represent the mean ± SEM of two or more independent experiments with three mice per group per experiment.  *P <  0.05 compared with control group; ^#^ *P* < 0.05 compared with HCl + vehicle group. Br: bronchus; V: venule.

We first evaluated the preventive effect of ellagic acid (10 mg/kg, p.o.) on the acid-initiated acute lung inflammation. The total cells, neutrophils, macrophages, and lymphocytes were quantified from the BALF. In nearly all of the time points analysed, the numbers of BALF total cells, neutrophils, macrophages, and lymphocytes from the vehicle-treated group were significantly increased compared to the saline control group (Figure 1(a)). The neutrophil numbers significantly increased after lung injury with maximal numbers at 12 h. Ellagic acid or dexamethasone treatment significantly reduced the total number of cells and neutrophils in the BALF compared to the vehicle-treated mice at 12, 48, and 72 h after injury (Figures 1(a) and 1(b)). The BALF neutrophil numbers of mice treated with ellagic acid were reduced by approximately 60%, 67%, and 73%, while the dexamethasone treatment reduced them by 78%, 50%, and 57% at 12, 48, and 72 h, respectively. Moreover, the ellagic acid significantly reduced the macrophages (45%) and the lymphocytes (87%) numbers at 48 h, while the dexamethasone only reduced the macrophages (38%) and the lymphocytes (60%) numbers at 72 h (Figure 1(d)). The histological analysis of the lung was performed at 12 h after injury. The lungs of the vehicle-treated mice demonstrated increased edema, *thickening* of the *alveolar septum* and interstitium, and leukocyte infiltration compared to the control group. Ellagic acid and dexamethasone treatment reduced all of the aforementioned inflammatory parameters compared to the vehicle-treated mice (Figure 1(e)).

### 4.2. Ellagic Acid Demonstrates a Therapeutic Effect

**Figure 2:**
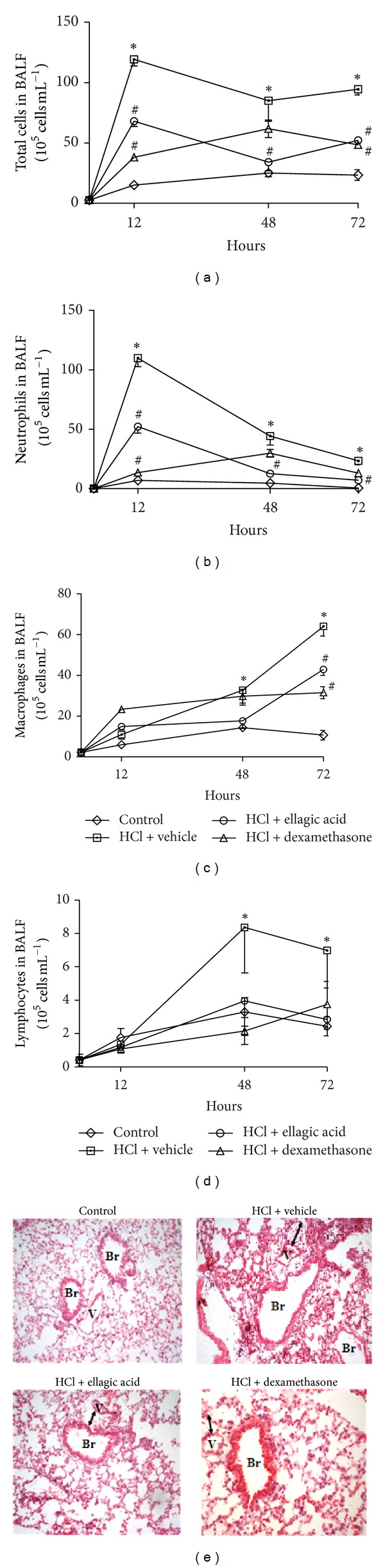
Ellagic acid demonstrates therapeutic anti-inflammatory activity in the experimental model acid-initiated acute lung injury. Mice received ellagic acid (10 mg/kg, p.o.), dexamethasone (1 mg/kg, s.c.), or vehicle (water, p.o.) after (2, 24, 47, and/or 71 h) the acid intratracheal instillation into the left lung (see Methods). The kinetics of inflammation was determined at 12, 48, and 72 hours after acid-initiated acute lung injury. (a) Total cells, (b) neutrophils, (c) macrophages, and (d) lymphocytes were evaluated in the BALF. One group of animals received saline intratracheal instillation (control group). (e) Lung tissue inflammation was assessed by hematoxylin and eosin staining (H&E, original magnification, 200x). Results represent the mean ± SEM of two or more independent experiments with three mice per group per experiment. *P < 0.05 compared with control group;  ^#^ *P* < 0.05 compared with HCl + vehicle group. Br: bronchus V: venule.

To investigate the therapeutic anti-inflammatory effect of ellagic acid on acid-induced ALI, the mice were treated with ellagic acid or the vehicle control at 2, 24, 47, or 71 h after lung injury. The numbers of total cells, neutrophils, lymphocytes, and macrophages in the vehicle-treated mice significantly increased compared to the saline control group in nearly all of the time points (Figure 2). The ellagic acid significantly reduced the numbers of total cells and neutrophils in the BALF compared to the vehicle-treated mice at all of the time points (12, 48, and 72 h) (Figures 2(a) and 2(b)). The dexamethasone treatment reduced the total cell numbers at 12 and 72 h and reduced the neutrophil numbers at 12 h (Figures 2(a) and 2(b)). The number of neutrophils in the BALF of animals that were treated with ellagic acid was reduced by approximately 52%, 71%, and 70% at 12, 48, and 72 h, respectively, while the dexamethasone treatment reduced the neutrophils by 87% at 12 h. The number of macrophages in the BALF was reduced by both ellagic acid (33%) and dexamethasone (51%) at 72 h (Figure 2(c)). No significant alteration was observed in the lymphocytes numbers by both ellagic acid and dexamethasone at all of the time points analyzed (Figure 2(d)). Additionally, ellagic acid and dexamethasone treatment reduced edema, thickening of the alveolar septum and interstitium, and leukocyte infiltration compared to the vehicle-treated mice (Figure 2(e)). 

### 4.3. Effect of Therapeutic Treatment with Ellagic Acid on the IL-6, IL-1*β*, KC, and IL-10 Levels in the BALF

**Figure 3:**
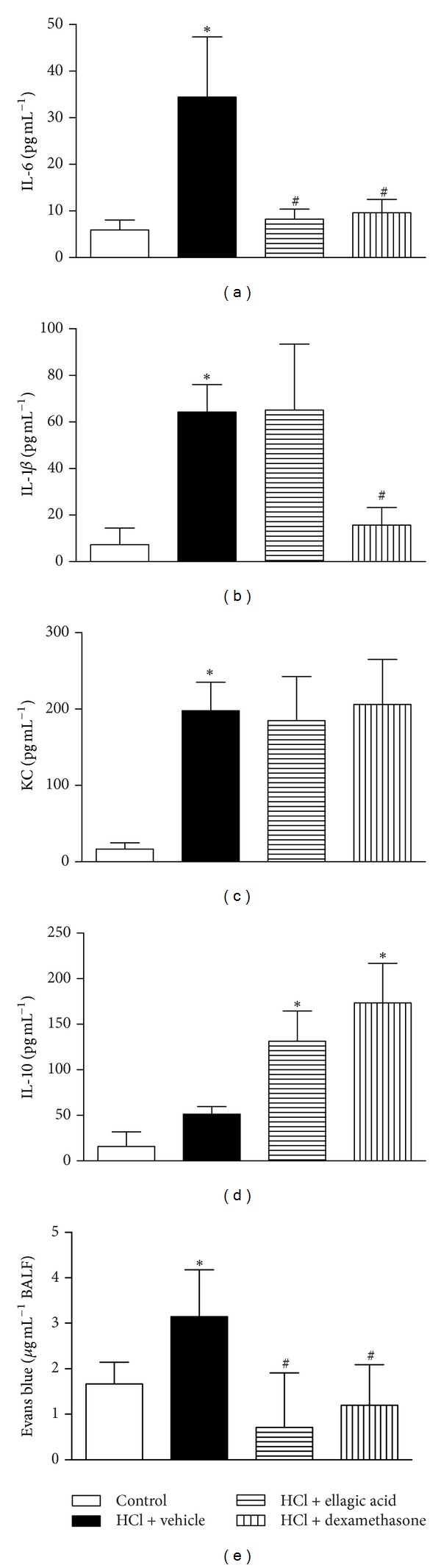
Effect of the therapeutic treatment with ellagic acid on IL-6 (a), IL-1*β* (b), KC (c), and IL-10 (d) levels in BALF. Evans Blue dye extravasation into BALF was also determined 12 h after ALI (e) (see Materials and Methods). Mice received in the therapeutic scheme ellagic acid (10 mg/kg, p.o.), dexamethasone (1 mg/kg, s.c.), or vehicle (water, p.o.) after (2 h) the acid intratracheal instillation into the left lung (see Methods). The analyses of lung were carried out in the peak of inflammation (12 h). Results represent the mean ± SEM of two or more independent experiments with three mice per group per experiment. *P < 0.05 compared with control group;  ^#^ *P* < 0.05 compared with HCl + vehicle group.

We next evaluated the therapeutic effect of ellagic acid on the BALF cytokine levels at 12 h after intratracheal administration of acid. The concentrations of IL-6, KC, and IL-1*β* were increased in the vehicle-treated mice compared to the saline control mice (Figures 3(a)–3(c)). Ellagic acid and dexamethasone treatment significantly reduced the IL-6 levels in the BALF (Figure 3(a)). IL-6 levels were reduced from 34.4 ± 7.4 pg/mL (vehicle control) to 11.369 ± 3.6 pg/mL (ellagic acid) and 13.3 ± 15.3 pg/mL (dexamethasone) (mean ± SEM). Treatment with dexamethasone, but not ellagic acid, reduced the BALF IL-1*β* concentration compared to the vehicle-treated mice (Figure 3(b)). In addition, neither ellagic acid nor dexamethasone reduced the KC levels compared to the vehicle control (Figure 3(c)). No significant alteration in IL-10 levels was observed in the vehicle-treated mice when compared to the saline control group (Figure 3(d)). Ellagic acid and dexamethasone treatment increased the levels of IL-10 by approximately eight- and elevenfold, respectively, compared to the saline control group (Figure 3(d)). 

The vascular permeability changes were increased in vehicle-treated mice compared to the saline control group. Ellagic acid and dexamethasone treatment also reduced the vascular permeability changes associated with ALI compared to the vehicle-treated mice (Figure 3(e)). 

### 4.4. Effect of Therapeutic Treatment with Ellagic Acid on Activator Protein 1 (AP-1) and Nuclear Factor Kappa B (NF-*κ*B) Activation and P-Selectin Expression in the Lung

**Figure 4:**
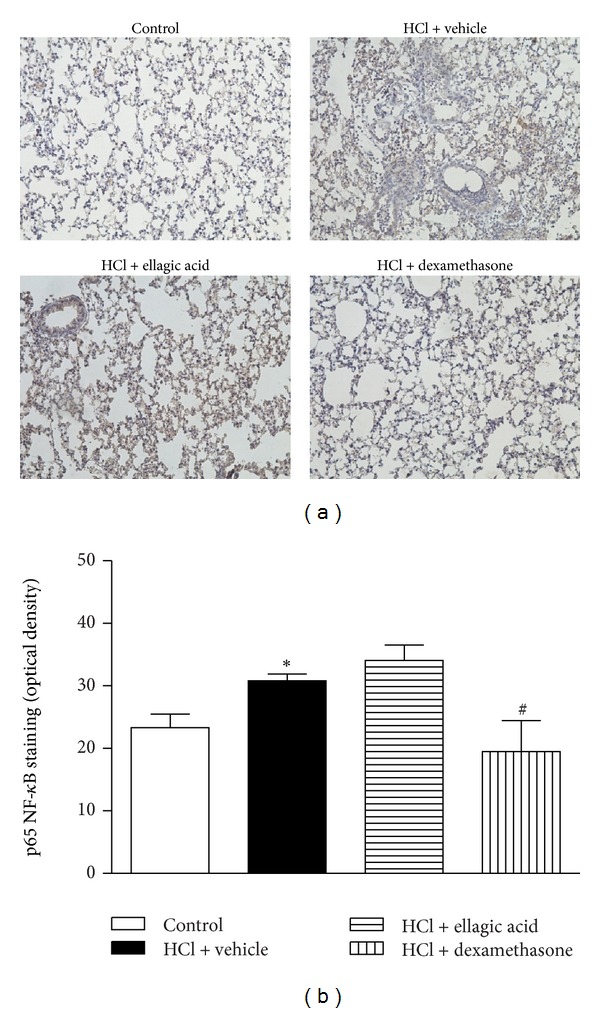
Effect of therapeutic treatment with ellagic acid on the activation of p65 NF-*κ*B. Representative images of phospho-p65 NF-*κ*B immunohistochemistry staining of control, HCl + vehicle group, HCl + ellagic acid group, and HCl + dexamethasone group (magnification: ×200) (a). The mean intensity of phospho-p65 NF-*κ*B staining was determined from image analysis and represented as arbitrary units (b). The analyses of lung were carried out in the peak of inflammation (12 h). Values represent the mean ± SEM (*n* = 4 per group) for immunohistochemical analysis. *P < 0.05 compared with control group;  ^#^ *P* < 0.05 compared with HCl + vehicle group.

**Figure 5:**
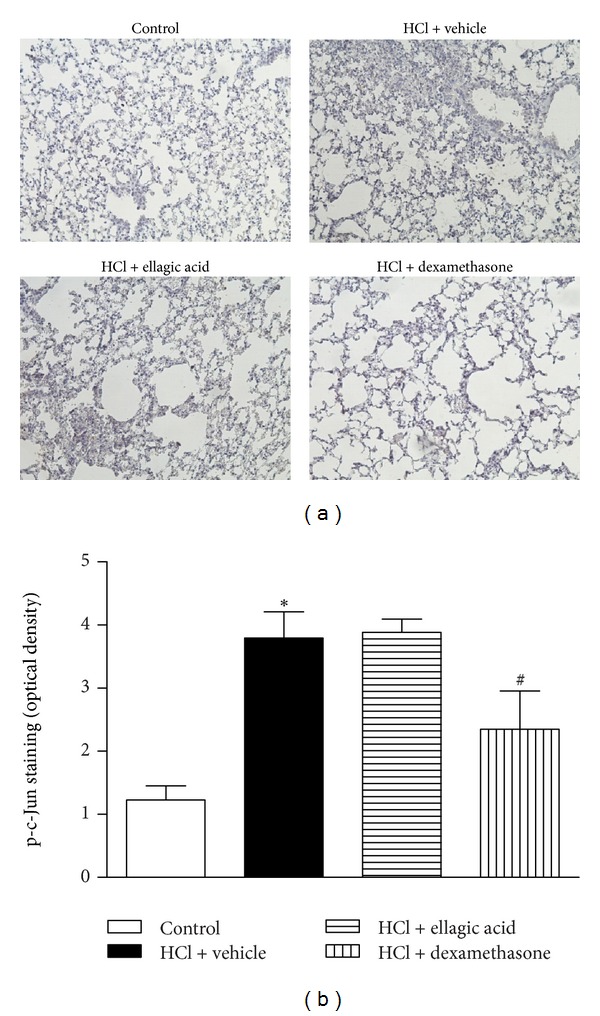
Effect of therapeutic treatment with ellagic acid on the activation of phospho-c-Jun AP-1. Representative images of phospho-c-Jun AP-1 immunohistochemistry staining of control, HCl + vehicle group, HCl + ellagic acid group, and HCl + dexamethasone group (magnification: ×200) (a). The mean intensity of phospho-c-Jun AP-1 staining was determined from image analysis and represented as arbitrary units (b). The analyses of lung were carried out in the peak of inflammation (12 h). Values represent the mean ± SEM (*n* = 4 per group) for immunohistochemical analysis. *P < 0.05 compared with control group;  ^#^ *P* < 0.05 compared with HCl + vehicle group.

We used immunohistochemistry to assess the effects of therapeutic ellagic acid treatment on P-selectin expression and on the phosphorylation state of AP-1 and NF-*κ*B at the peak of lung inflammation (12 h). p65 NF-*κ*B staining was observed in the nucleus of the lung cells from the saline control mice. A discrete activation of c-Jun was also observed in the control group. p65 NF-*κ*B and c-Jun activation was observed in the bronchial epithelium of the vehicle-treated mice (Figures 4 and 5) compared to the control group. Treatment with dexamethasone, but not ellagic acid, decreased the activation of p65 NF-*κ*B and c-Jun compared with the vehicle-treated mice (Figures 4 and 5). P-selectin staining was observed in all groups; however, no significant alteration in staining was observed among the groups (data not shown). 

### 4.5. The Anti-Inflammatory Effect of Ellagic Acid on the COX-2 Inhibition-Induced Exacerbation of Inflammation

**Figure 6:**
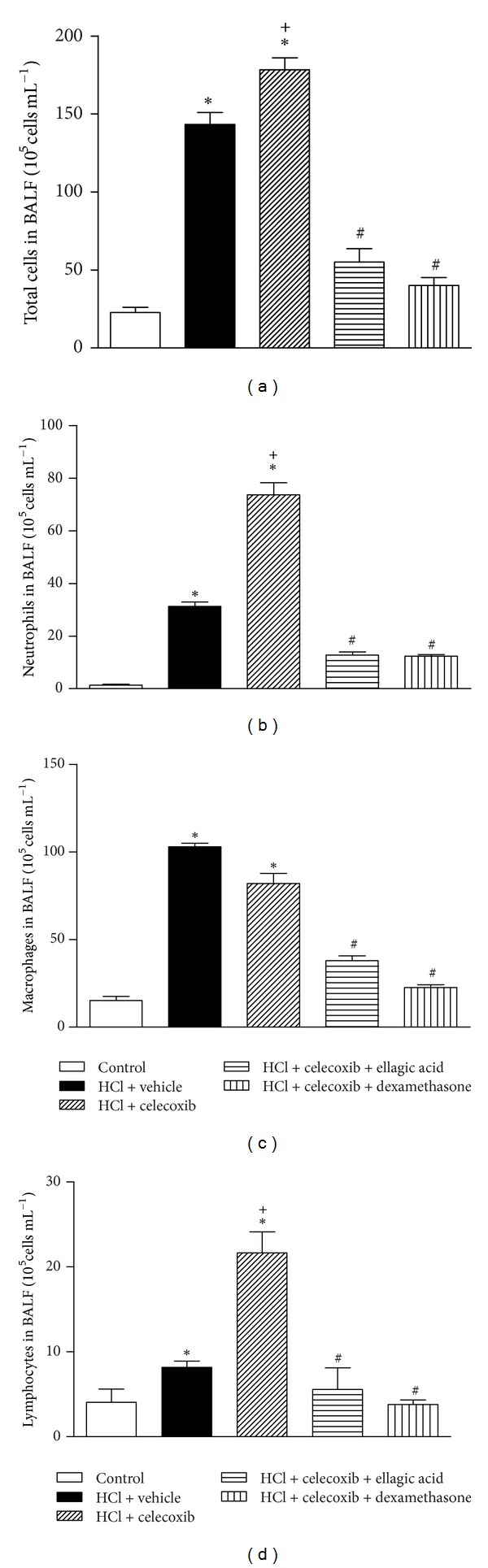
Ellagic acid (therapeutic treatment) dampens the exacerbation of inflammation induced by COX-2 inhibition in experimental model of acid-induced acute lung injury. Mice received ellagic acid (10 mg/kg, p.o.), dexamethasone (1 mg/kg, s.c.), or vehicle (water, p.o.) after (2, 24, and/or 47 h) the acid intratracheal instillation into the left lung. (a) Total leukocytes, (b) neutrophils, (c) macrophages, and (d) lymphocytes were evaluated in the BALF at 48 h after the acid instillation. Results represent the mean ± SEM of two or more independent experiments with three mice per group per experiment. *P < 0.05 compared with control group;  ^+^ *P* < 0.05 compared with HCl + vehicle group; ^#^ *P* < 0.05 compared with HCl + celecoxib group.

We next assessed the therapeutic effect of ellagic acid on the exacerbated inflammatory response of mice exposed to experimental acid-induced ALI and treated with the selective COX-2 inhibitor (celecoxib, 10 mg/kg; i.p.) at 48 h. Compared to the saline control group, the vehicle- or the celecoxib-treated mice displayed increased numbers of total cells, neutrophils, macrophages, and lymphocytes in the BALF (Figure 6). In addition, celecoxib-treated mice had increased total cells, neutrophils, and lymphocytes in the BALF when compared to the vehicle-treated mice (Figure 6). Ellagic acid (10 mg/kg) and dexamethasone (1 mg/kg) reduced the exacerbation of ALI inflammation induced by COX-2 inhibition. Both treatments reduced the recruitment of total cells, neutrophils, macrophages, and lymphocytes into the BALF. Ellagic acid and dexamethasone treatment reduced the number of total cells from 178.3 ± 3.2 (selective inhibitor of COX-2) (mean × 10^5^ cells/mL ± SEM) by approximately 69% and 77%, respectively, to 55.0 ± 3.2 (mean × 10^5^ cells/mL ± SEM) and 40.0 ± 1.8 (mean × 10^5^ cells/mL ± SEM) (Figure 6(a)). Similarly, ellagic acid and dexamethasone treatment reduced the number of neutrophils from 73.7 ± 6.4 (selective inhibitor of COX-2) (mean × 10^5^ cells/mL ± SEM) by approximately 82% and 83%, respectively, to 12.7 ± 1.6 (mean × 10^5^ cells/mL ± SEM) and 12.3 ± 1.0 (mean × 10^5^ cells/mL ± SEM) (Figure 6(b)). The number of macrophages was reduced from 82.0 ± 6.5 (selective inhibitor of COX-2) by approximately 53% and 72% to 37.9 ± 3.3 (ellagic acid) (mean × 10^5^ cells/mL ± SEM) and 22.5 ± 2.8 (dexamethasone) (mean × 10^5^ cells/mL ± SEM), respectively (Figure 6(c)). The number of lymphocytes was reduced from 21.6 ± 4.0 (selective inhibitor of COX-2) by approximately 75% and 82% to 5.5 ± 13.6 (ellagic acid) (mean × 10^5^ cells/mL ± SEM) and 3.8 ± 2.8 (dexamethasone) (mean × 10^5^ cells/mL ± SEM), respectively (Figure 6(d)).

### 4.6. Effect of ellagic Acid on the Resolution Phase of Inflammation

**Figure 7:**
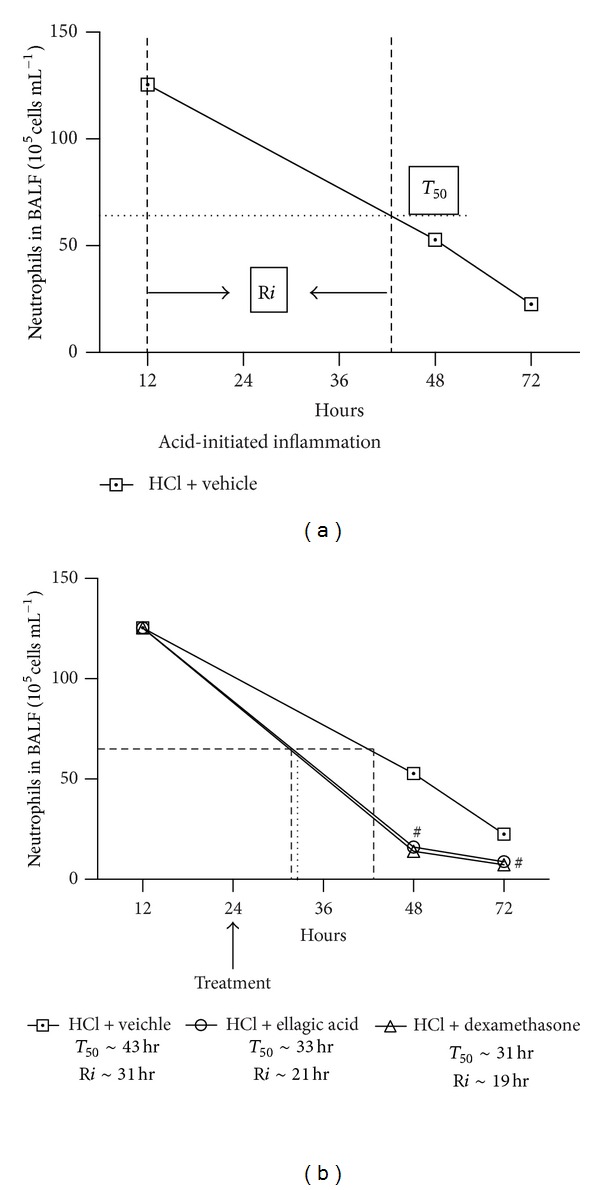
Ellagic acid accelerates the resolution of inflammation of acid-initiated acute lung injury. *T*_50_ correspond to the time point to reduce ~50% of neutrophils. The resolution interval (R*i*) is defined as the time required for cell numbers to decrease to 50% of the maximum at peak inflammation (the interval between peak of inflammation (at 12 h) and *T*_50_). These indices were calculated when ellagic acid, dexamethasone, or vehicle were administrated in the resolution phase at 12 h after the peak of inflammation (24 h). Results represent the mean ± SEM of two or more independent experiments with three mice per group per experiment. *P < 0.05 compared with control group;  ^#^ *P* < 0.05 compared with HCl + vehicle group.

As our results suggested a protective effect of ellagic acid on the airway, we next determined the influence of ellagic acid on the resolution of established airway inflammation. *T*_50_ correspond to the time point to reduce ∼50% of neutrophils, and resolution interval (R*i*) is defined as the time required for the cell numbers to decrease to 50% of the peak of inflammation (the interval between peak of inflammation, at 12 h, and *T*_50_) [22, 23] (Figure 7(a)). In the vehicle-exposed mice, the *T*_50_ was ∼43 h, and the endogenous resolution interval for the BALF neutrophils was ∼31 h. The treatment with ellagic acid, vehicle, and dexamethasone was carried out 12 h (in the resolution phase) after the peak of inflammation (12 h). The *T*_50_ and R*i* for the BALF neutrophils were markedly decreased with the ellagic acid treatment to ∼33 h and ∼21 h (∼67% of the vehicle resolution interval), respectively (Figure 7(b)), indicative of more rapid resolution of acute inflammation. The dexamethasone treatment also decreased the *T*_50_  and R*i* to ∼31 h and ∼19 h (∼61% of the vehicle resolution interval) (Figure 7(b)), respectively.

## 5. Discussion

In the present study, the ellagic acid displayed anti-inflammatory properties by decreasing the severity of HCl acid-initiated ALI, accelerating the resolution of inflammation and decreasing the COX-2 inhibitor-induced exacerbation of inflammation. Ellagic acid reduced several inflammatory parameters, including the vascular permeability alterations and the neutrophil recruitment to the BALF and the lung. In addition, ellagic acid reduced the proinflammatory cytokine IL-6 and increased the anti-inflammatory cytokine IL-10 in the BALF without downregulating the NF-*κ*B and AP-1 signaling pathways (different from dexamethasone). Together, these findings demonstrated that ellagic acid has potential anti-inflammatory effects for the resolution of ALI inflammation.

Although inflammation is essential for the maintenance of tissue homeostasis and protection against infection, uncontrolled and persistent inflammation may contribute to tissue damage, a characteristic phenomenon of several inflammatory disorders, including ALI. In airway inflammation of ALI, the neutrophils are the first cells to be recruited and are the predominant cause of tissue damage [24]. A persisting neutrophilia is associated with a poor outcome of ALI [25]. In addition, another hallmark of ALI is edema, which is a consequence of the increased permeability of the alveolar-capillary barrier, as well as epithelial damage, which results in the impairment of arterial oxygenation [24, 26]. Over the past several years, no therapeutic agents have demonstrated a clear benefit during ALI treatment [4, 27]. 

In the continued search for bioactive plant-derived products, several groups, including our own, have successfully employed experimental models to screen the pharmacologic activities of plant extracts, as well as isolated compounds, such as ellagic acid [11]. Utilising secondary metabolites from medicinal plants that can control leukocyte recruitment, such as neutrophils, could be a strategy to reduce the lung damage during ALI. Phytochemical and pharmacological studies have identified many potential anti-inflammatory substances, particularly those derived from plants used in folk medicine. *Lafoensia pacari* (Lythraceae) has been used in traditional medicine to treat gastric ulcers and inflammation in the state of Mato Grosso (Brazil). In a bioassay-guided fractionation of the *Lafoensia pacari* extract, Rogerio et al. [11] identified ellagic acid as the compound responsible for the reduction of neutrophil recruitment into the peritoneal cavity of mice induced to injury by *Histoplasma capsulatum*-derived *β*-glucan. This reduction in the recruitment of neutrophils and eosinophils to the BALF by ellagic acid has also been observed in an ovalbumin-induced experimental allergic airway inflammation model [15]. In addition*, Lafoensia pacari* extract and ellagic acid demonstrated antiedematous activity in a mouse paw edema model [11, 28]. 

Ellagitannins present in the pomegranate (*Punica granatum*) fruit, which has been used for centuries for medical purposes. Studies with pomegranate extract have demonstrated its anti-inflammatory effects in murine models of collagen-induced arthritis [29] and experimental colitis [30, 31]. In an experimental model of ALI (LPS-initiated), the pomegranate extract also reduced the myeloperoxidase (a heme enzyme present in the primary granules of neutrophils) in the lungs of mice [17]. 

The free ellagic acid is absorbed in the gut, but its bioavailability is low [32]. It binds in the intestinal epithelial cells [33], and its uptake might occur through monolayer of these cells [34]. In addition, ellagic acid could be conjugated (sulphate ester, glucuronide, and glutathione conjugates) [33, 35] or metabolized by bacteria in the gut to produce urolithins (A and B) [36]. These microbial metabolites are more bioavailable than ellagic acid, and in the human plasma, they were detected after consuming pomegranate extract capsule (21.6 mg of free ellagic acid) at 8 and 24 h [28]. So, these metabolites could be also responsible for the biological activity of ellagic acid such as anti-inflammatory activity [29, 37]. Interesting, while these metabolites do not accumulate in organ tissues [38], the free ellagic acid irreversibly binds to macromolecules (proteins and DNA) [34] and consequently might accumulate in the epithelial cells and others cells. Ellagic acid was detected in the lung of mouse after oral administration (at dose 2.0 mmol  ~6 mg/kg) [39]. In addition, these authors demonstrated that ellagic acid localized preferentially in lung (10-fold higher) when compared to liver. So, if beneficial effects of ellagic acid such as the anti-inflammatory activity observed by us in the acute lung injury are associated with urolithin production is not possible to answer. Further studies are needed to analyze this hypothesis. So, as ellagic acid, urolithins, or both could be responsible by this activity, however, only ellagic acid is detected in the lung, and because of this, probably the ellagic acid is major responsible for reducing the airways inflammation.

The key result from our study was that the ellagic acid demonstrated both preventive and therapeutic effects in reducing edema and leukocyte recruitment to the BALF, similar to dexamethasone, during ALI. In addition, ellagic acid, similar to dexamethasone, accelerated the resolution of inflammation by promoting the recovery from lung injury and reduced the exacerbation of inflammation induced by COX-2 inhibition [18]. These results suggest that ellagic acid has potent anti-inflammatory activity and is able to reduce the airway inflammation, as well as its exacerbation, during ALI. 

It is well known that adhesion molecules are involved in leukocyte influx. Selectins, a family of transmembrane molecules that are expressed on the surface of leukocytes and activated endothelial cells, are critically involved in leukocyte recruitment. We evaluated the expression of P-selectin in the lungs, as this molecule is considered to be an important target for modulating neutrophil influx into the inflamed tissue [40, 41]. Our findings revealed no significant alterations in the expression of P-selectin in the bronchial epithelium among the groups (data not shown).

Inflammatory diseases are controlled by proinflammatory transcription factors, such as NF-*κ*B and AP-1 [42]. Eickmeier et al. [43] demonstrated that NF-*κ*B expression and nuclear translocation increased in airway epithelial cells in an ALI model (acid-initiated). Unlike dexamethasone treatment, ellagic acid did not reduce NF-*κ*B activation during the peak of inflammation (12 h) in acid-initiated ALI. In addition, only dexamethasone treatment reduced AP-1 activation. These findings suggest that the effect of ellagic acid on acid-induced ALI was NF-*κ*B and AP-1 independent. 

In ALI, a complex network of cytokines and chemokines, such as IL-6, IL-1*β*, and KC, among others, mediate the inflammatory response. Several studies have demonstrated that ellagic acid can inhibit cytokines and chemokines *in vivo* and *in vitro* [14, 44]. Of particular interest, ellagic acid, similar to dexamethasone, reduced the levels of IL-6 in the BALF of mice with acid-initiated ALI. In mice with LPS-initiated ALI, IL-6 was correlated with a proinflammatory phenotype [45], and high levels of IL-6 in the plasma and BALF of humans were associated with an increased risk of developing ALI [24]. IL-10 is an anti-inflammatory cytokine with a significant role in preventing inflammatory diseases [46]. IL-10 demonstrated a protective role in LPS-induced ALI [24]. In the acid-initiated ALI, ellagic acid, similar to dexamethasone, increased the IL-10 concentration in the BALF. Dexamethasone, unlike ellagic acid, did not reduce the IL-*β* concentrations. Neither dexamethasone nor ellagic acid reduced the KC concentration in the BALF. Therefore, these results suggest that ellagic acid could improve the outcome, as well as the prognosis, of ALI in patients by modulating the cytokines involved in ALI physiopathology. However, further studies are needed before this possibility can be examined in humans. 

In conclusion, ALI is a disease with high morbidity and mortality, and currently, the disease outcome has yet to be improved by pharmacologic treatment. Ellagic acid has demonstrated antioxidant and anti-inflammatory activity in several *in vivo* and *in vitro* experimental inflammation models. In HCl acid-initiated ALI, ellagic acid demonstrated potent anti-inflammatory effects, accelerated the resolution of inflammation, and decreased the exacerbation of the inflammation process caused by selective COX-2 inhibition. These effects were due to the modulation of cytokines (decrease of IL-6 and increase of IL-10 in the BALF). The inability of ellagic acid to modulate the NF-*κ*B and AP-1 signaling pathways suggests that this molecule may control inflammation without inducing an immunosuppressive response, as is observed during dexamethasone treatment. In addition, the consumption of pomegranate juice, which contained 121 mg/L of ellagic acid, did not produce toxic effects in humans over a 3-year period [47]. Moreover, the *L. pacari* extract (which contains ellagic acid) did not show cytotoxicity *in vitro* or *in vivo* [48]. In conclusion, ellagic acid might be a future alternative treatment for the reduction of inflammation during ALI and other inflammatory diseases with fewer adverse effects than corticosteroids.
